# High Quality Targeted Temperature Management (TTM) After Cardiac Arrest

**DOI:** 10.1186/s13054-019-2721-1

**Published:** 2020-01-06

**Authors:** Fabio Silvio Taccone, Edoardo Picetti, Jean-Louis Vincent

**Affiliations:** 1 Department of Intensive Care, Cliniques Universitaires de Bruxelles Hopital Erasme, Université Libre de Bruxelles (ULB), Route de Lennik 808, 1070 Brussels, Belgium; 2 Department of Anesthesia and Intensive Care, Parma University Hospital, Parma, Italy

**Keywords:** Temperature, Cardiac arrest, Quality, Outcome

## Abstract

Targeted temperature management (TTM) is a complex intervention used with the aim of minimizing post-anoxic injury and improving neurological outcome after cardiac arrest. There is large variability in the devices used to achieve cooling and in protocols (e.g., for induction, target temperature, maintenance, rewarming, sedation, management of post-TTM fever). This variability can explain the limited benefits of TTM that have sometimes been reported. We therefore propose the concept of “high-quality TTM” as a way to increase the effectiveness of TTM and standardize its use in future interventional studies.

## Background

Post-anoxic brain damage is the most dramatic complication of cardiac arrest [1]. In international guidelines, targeted temperature management (TTM) is the only neuroprotective intervention currently recommended after out-of-hospital cardiac arrest (OHCA) [2]. Nevertheless, the scientific community has raised concerns about the level of evidence supporting this recommendation [3]. Two early randomized clinical trials (RCTs) showed that TTM at 33 °C for 12–24 h was associated with a greater proportion of survivors with intact neurological recovery compared to standard care in OHCA survivors with witnessed shockable rhythm [4, 5], but subsequent observational studies questioned the efficacy of this intervention in other settings, such as non-shockable rhythms and in-hospital cardiac arrest (IHCA) [6, 7]. After publication of the so-called “TTM trial” in 2013, which showed similar survival and neurological recovery rates in OHCA patients treated at 33 °C or at 36 °C for 24 h [8], the use of TTM decreased significantly [9, 10] as many physicians considered that keeping patient body temperature within normothermic ranges (i.e., at about 37 °C) would likely be as effective as using TTM at 36 °C, without the adverse events related to cooling procedures, including use of sedative drugs.

Many “supporters” of TTM criticized the “TTM trial” [8], emphasizing that a number of features, including the high patient heterogeneity, the very short time to resuscitation, the slow induction phase of TTM, and the rapid rewarming period, may have influenced the main results, and still consider TTM at 33 °C as the best therapeutic option in cardiac arrest survivors. This position is supported by the publication of the recent HYPERION study, which showed a significant improvement in neurological outcome at 3 months for patients with OHCA or IHCA associated with a non-shockable initial rhythm who were treated with TTM at 33 °C, compared to a control group kept at 37 °C [11]. Although some of the criticisms of the “TTM trial” may have been reasonable, it was the largest study in this field and was conducted using sound up-to-date statistical methodology [8]. Moreover, looking at the early evidence supporting the use of TTM, one can argue that populations were highly selected and results were not generalizable to all cardiac arrest victims. These early studies also had many methodological biases (e.g., no power calculation, relatively small cohorts, early stopping because of lack of funding, no blinded assessors of primary outcome, no prognostication guidelines) and the control group experienced fever (i.e., temperature > 38 °C), which might have been responsible for detrimental effects, thus overestimating the beneficial effects of TTM [4, 5]. Importantly, the significant improvement in the clinical management of cardiac arrest patients (including early coronary angioplasty, standardized hemodynamic and ventilatory targets, avoidance of early withdrawal of life-sustaining therapies) between the early trials [4, 5] and the “TTM trial” [8] may in part explain the blunted effects of TTM at 33 °C in this study. Finally, the enthusiastic results supporting the effectiveness of TTM in experimental cardiac arrest [12] may not be directly translated in humans because the animals used in these models do not have comorbidities and/or underlying cardiac disease; resuscitation is standardized and cooling is immediate; the brain size is smaller; and some measured outcomes only included histological lesions and/or biomarkers of brain injury, which cannot reflect “cognitive function” as assessed in human studies.

Today, when treating patients resuscitated after cardiac arrest, the medical community is separated into TTM “believers” and “neutral,” with a significant impact on patient management and a trend towards a less accurate TTM prescription or, in the worst scenario, a “nihilistic” approach, with the total abandon of any temperature control in a number of centers.

## The concept of “high-quality TTM”

**Fig. 1:**
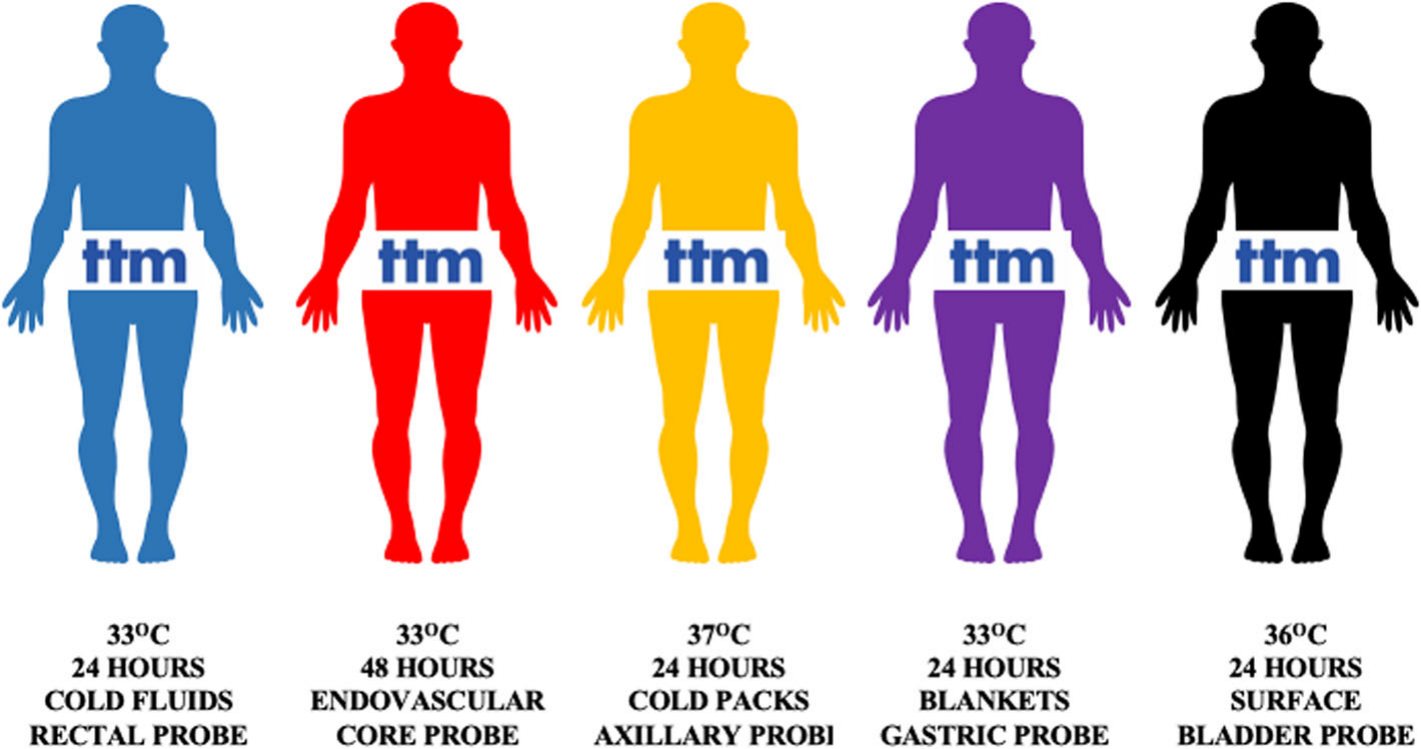
Different approaches to providing targeted temperature management (TTM), regardless of its quality, in five hypothetical patients admitted after successfully resuscitated cardiac arrest. Colors identify different patients and do not refer to quality of TTM

When prescribing a drug, physicians consider its mechanism of action and the appropriate route (oral or intravenous), dose, and duration, according to specific information collected from clinical trials. As an example, to compare two anti-inflammatory agents for pain relief, the patients randomized into the two study arms will receive the regimens that would result in the most potent anti-inflammatory effects for both molecules. Unfortunately, this “most effective” protocol for TTM is undefined. We could have, for example, five OHCA survivors admitted to five different intensive care units (ICUs), who could receive different TTM protocols, as indicated in Fig. 1; despite the differences in treatment modalities and targets, they would all be included and considered in the “TTM group” of a pragmatic multicenter RCT, thus adding significant heterogeneity to the delivery of TTM and its effects on outcome.

Returning to our analogy with drugs, we need to define the optimal way of delivering TTM, specifying the characteristics that could provide the best neuroprotective effects after anoxic brain injury with minimal adverse effects. This approach is also similar to the concept of “high-quality cardiopulmonary resuscitation (CPR)” [13], which considers the correct rate and depth of compression, with minimal interruptions, to increase the probability of success. As such, “high-quality TTM” should be considered in clinical protocols when TTM is initiated.

## How to define “high-quality TTM”?

In 2009, a consensus of five scientific societies introduced the concept of “targeted temperature management” to replace the previous term of “therapeutic hypothermia” [14], to underline the clinical relevance not only of the cooling or maintenance period, but also of the other phases of therapy, including induction, rewarming, and normothermia. However, we still lack good clinical data and knowledge about the optimal method, including when best to initiate TTM, the target temperature, the duration, and the rewarming rate. The “TTM study” from Nielsen et al. [8] only investigated the most effective target temperature but did not explore the other questions related to optimal TTM. We will summarize the current evidence for each aspect in the next sections. The discussion will not include the selection of the patients who would benefit the most from TTM, which is a relevant and unresolved issue, but beyond the scope of the viewpoint.

### Timing of initiation

TTM should be initiated as soon as possible to minimize reperfusion injury following the return of spontaneous circulation after cardiac arrest [15]. However, two RCTs showed that pre-hospital cooling using intravenous cold fluids did not improve outcomes and was associated with more early re-arrests and more pulmonary edema on hospital admission than no pre-hospital cooling [16, 17]. Similar results were obtained when cold fluids were administered during CPR (intra-arrest TTM), which theoretically should have even greater beneficial effects on the anoxic brain [18, 19]. However, the use of intra-arrest TTM using a trans-nasal device, which is a method able to primarily induce brain cooling during CPR, showed some potential benefits, in particular in OHCA victims with an initial shockable rhythm [20, 21], suggesting that the method used to induce intra-arrest TTM may be determinant in maximizing brain protection and avoiding adverse effects.

### Temperature measurement

Immediately after the decision to initiate TTM, body temperature should be measured using a probe placed in the bladder, the esophagus, or a vessel (artery or vein). This approach is the most accurate to assess the “core” temperature, which gives the closest approximation to brain temperature, although brain temperature may be 0.4 to 2.0 °C higher than core temperature after acute brain injury [22]. Other methods, such as oral probes and infrared ear or axillary thermometry, should be avoided. Rectal temperature changes with some delay when compared to the core one so that rectal probes should not be used [23]. Moreover, body temperature should be measured continuously in all patients; intermittent recording of body temperature may miss large fluctuations in temperatures and result in inappropriate TTM delivery.

### Target temperature

The target body temperature should be 33 °C or 36 °C, as it has been studied in the “TTM trial.” Also, another target could be selected between these two ranges (i.e., 34 °C); nevertheless, whatever the final decision, it is important to strictly control the target temperature at the selected value. In one study, when TTM was strictly maintained at target values using a surface method with temperature feedback to constantly adjust the intensity of cooling, there was no difference in patient outcomes between a target of 33 °C and one of 36 °C [24]. However, in a before-after study comparing change in TTM target from 33 to 36 °C, there was poor compliance with the higher target temperature resulting in reduced times in target and increased fever rates; there was also a 15% reduction in patients with favorable neurological outcome compared to the earlier period [25]. Similar results were observed in other studies [26, 27]. It remains still unclear whether some patients may benefit more from one or the other target temperature. One may argue that the higher target temperature (i.e., 36 °C) could be preferable in patients with an increased risk of adverse events at lower temperatures, e.g., with bleeding or severe hemodynamic impairment, whereas the lower target (i.e., 33 °C) may be preferred in patients with greater risk of neurological damage, as suggested by prolonged CPR, occurrence of seizures, or evidence of cerebral edema on brain imaging, which may be worsened by higher temperatures.

### Duration of the cooling phase

The cooling phase should last at least 24 h. A RCT showed no difference in neurological outcomes between TTM at 33 °C for 24 or 48 h, although the longer duration was associated with a 5% improvement in favorable long-term neurological outcome [28]. Considering the promising results from prolonged (i.e., 72 h) cooling in newborns [29] and the absence of increased complications from TTM given over 48 h [28], no data support the use of short duration TTM in adult cardiac arrest victims, which should be then avoided.

### Duration of the rewarming phase

The rewarming rate should be slow (0.15–0.25 °C/h) and controlled using specific TTM devices, rather than spontaneous, which may result in unpredictable rewarming speeds. Recent small and highly biased studies have suggested that a slow and controlled rewarming rate is feasible and may be associated with better long-term neurological outcomes [30, 31]. Finally, because many studies have suggested a detrimental effect of post-TTM fever on outcome [32, 33], careful control of body temperature for at least 48 h following the end of rewarming is mandatory; this strategy could be titrated to patient condition (i.e., shorter if immediate awakening or prolonged in case of signs of moderate to severe cerebral insult).

## How to achieve “high-quality TTM”?

The definition and the methods to achieve high quality of TTM are intrinsically linked. To achieve precise and accurate TTM, two issues are crucial: the use of sedatives/analgesics and the choice of device.

### Pharmacologic interventions

Sedative and analgesic agents should be used in all cardiac arrest patients undergoing TTM. These drugs contribute to reduce shivering, which is responsible for heat generation and results in a prolonged time to target temperature, high temperature variability during the maintenance phase, and faster rewarming [34]. No study has shown superiority of any particular sedation regimen on patient outcome; however, the use of short-acting drugs (e.g., propofol and remifentanil) may limit drug accumulation and delayed awakening, although propofol may result in more hemodynamic disturbance [35]. Regardless of the sedative and analgesic regimen chosen, drugs should be administered at the time of TTM initiation and discontinued only at normothermia (i.e., 37 °C). In case of shivering during the normothermia phase, analgesics and low-dose sedatives, together with intravenous magnesium and α_2_-agonists, should be used [36]. Commonly used antipyretic drugs, such as paracetamol or non-steroidal anti-inflammatory drugs, are of limited effect during the induction and maintenance phase, while they might be useful at normothermia to avoid or minimize fever, as adjunctive therapies.

Finally, adjunction of neuromuscular blocking agents, either as a bolus or continuous infusion [37], is very effective in the induction of TTM to enable the target temperature to be reached rapidly and may be useful during the maintenance and rewarming phases to avoid temperature variation, in particular in patients receiving very high doses of sedatives and analgesics, which may have relevant adverse effects, or shivering refractory to other pharmacologic interventions. To adequately detect early shivering, some scale, such as the Bedside Shivering Assessment Scale (BSAS), could be of interest in managing these patients [38].

### Device selection

Among the various methods that exist to provide TTM, automated devices using a temperature feedback system (TFS) provide a more rapid time to target temperature, less temperature variability, and accurate and slow rewarming compared to external methods, such as ice packs, ice pads, or cold fluids. In a recent systematic review, Calabró et al. showed that, although the literature consisted largely of retrospective or prospective studies, the use of core and/or TFS devices was associated with a lower probability of poor neurological outcome when compared to other methods [39]. Although non-automated methods are cheaper and easier to apply, temperature control is poor and their use should be limited to the induction phase in combination with automated devices.

## Perspectives and conclusions

**Fig. 2:**
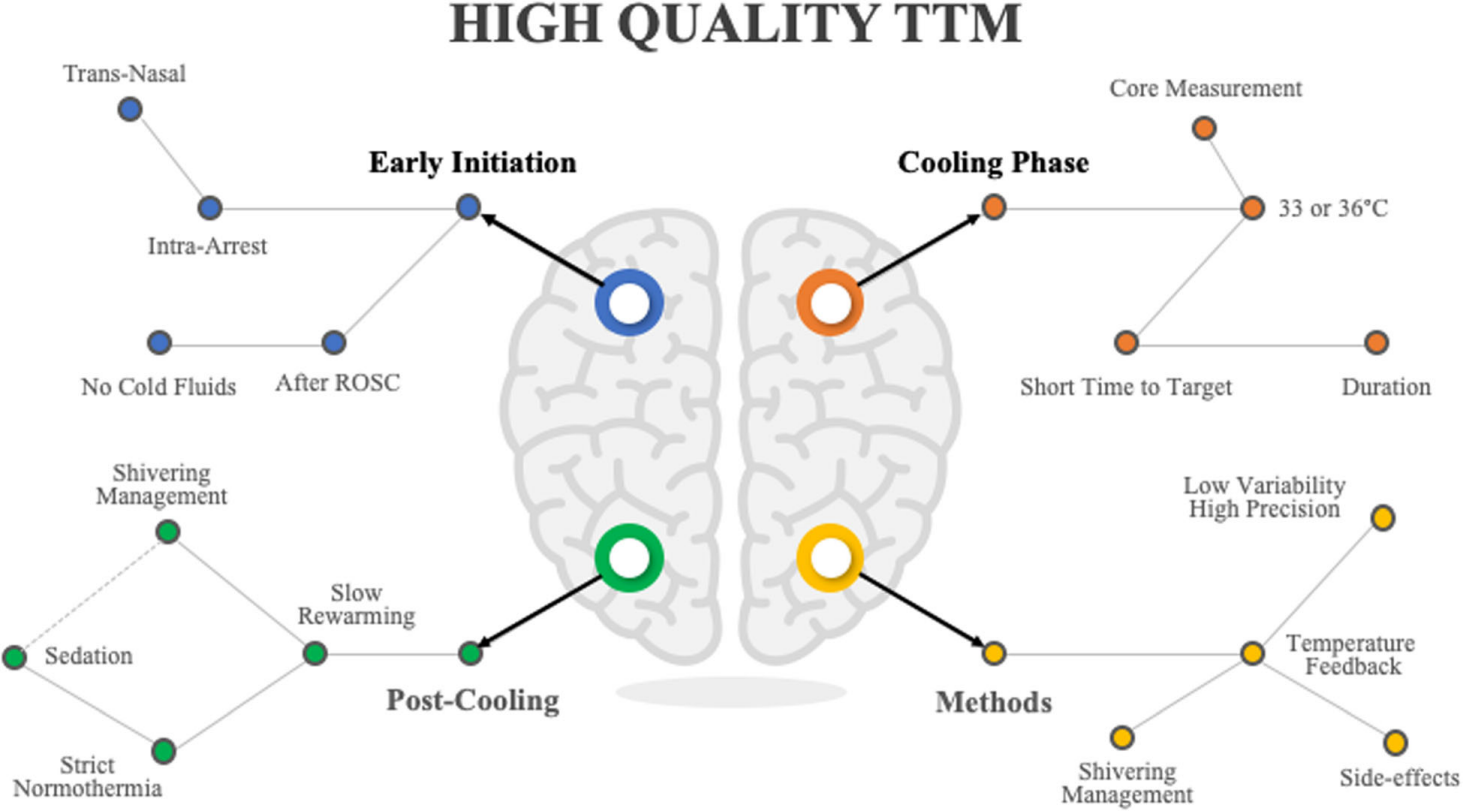
Some of the various factors related to targeted temperature management (TTM) which are relevant to providing “high-quality” TTM

**Fig. 3:**
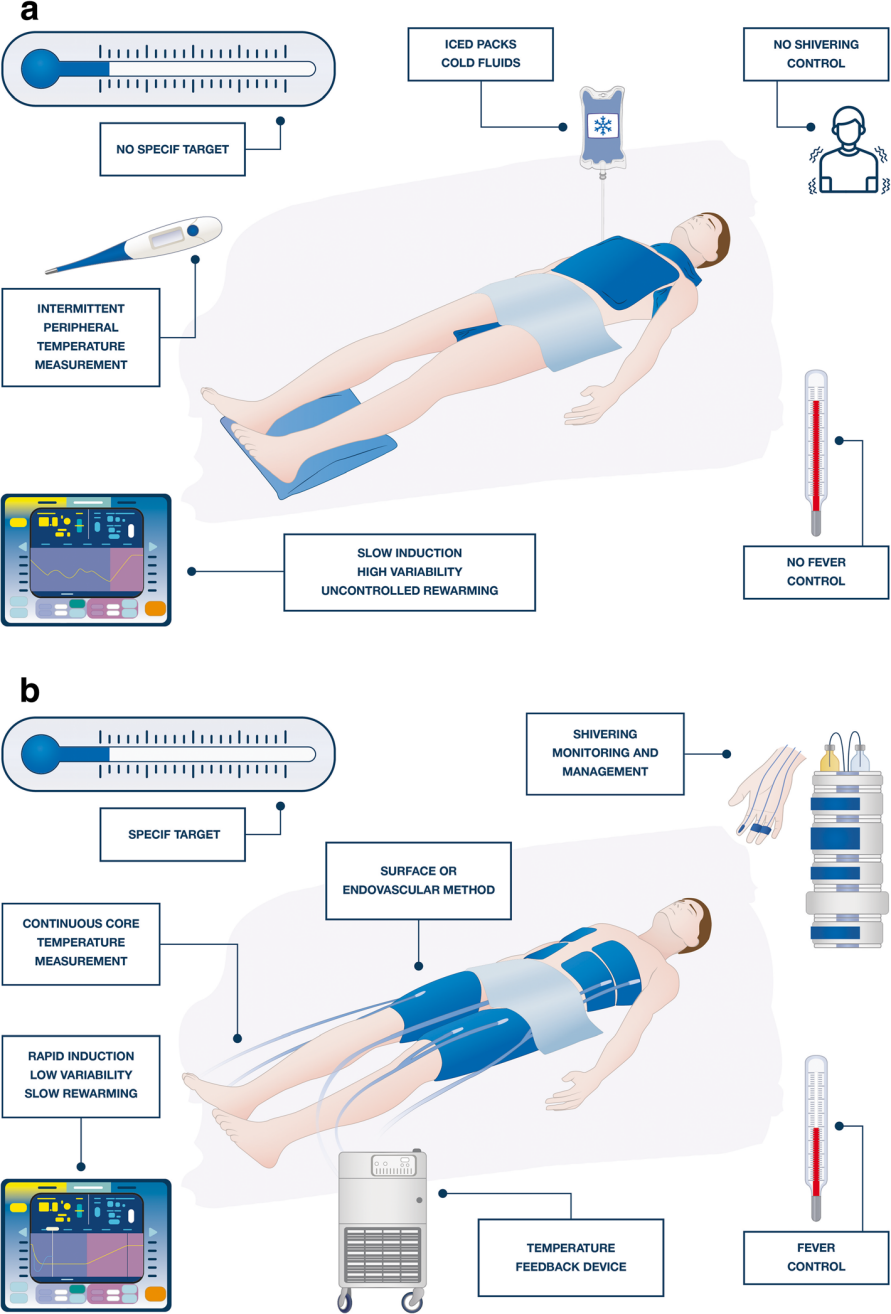
Practical representation of “low-quality” (**a**) and “high-quality” (**b**) targeted temperature management (TTM) in a patient resuscitated and admitted to the hospital after cardiac arrest. The colors of the screen and temperature time course as of the TTM methods (blankets or external device) are not intended to promote any specific cooling device

TTM is a complex therapy and requires a “bundle” of interventions and clinical decisions that require standardization in order to maximize their neuroprotective effects (Fig. 2). As such, protocols should be used in clinical practice to improve the homogeneity of TTM prescription; these protocols could result in an improvement in patients’ outcome [40]. If body temperature and shivering are not adequately monitored, induction is delayed, body temperature remains variable using non-automated methods, and spontaneous and fast rewarming are allowed, the patient will be exposed to “low-quality TTM” and the likelihood of a beneficial effect will be compromised (Fig. 3a). By contrast, early initiation after the anoxic injury, continuous core temperature monitoring, the use of drugs to facilitate the decrease in temperature and prevent shivering, the selection of a specific target temperature during the cooling phase, a maintenance phase with well-regulated constant temperature, a prolonged rewarming phase, and the avoidance of fever after TTM are the main components of “high-quality TTM” (Fig. 3b). It is interesting to note that this “high-quality TTM” has been used in several experimental studies [15] and may explain why marked neuroprotective effects could be obtained with only a few included animals, whereas “low-quality TTM” has often been observed in large pragmatic RCTs [8].

Before clinical studies can finally validate the clinical significance of this concept on measured relevant outcomes, future studies should further explore TTM characteristics, such as target temperature or duration) while ongoing studies will provide relevant information in the next years; one ongoing RCT will evaluate whether TTM at 33 °C for 24 h is more effective than early treatment of fever (i.e., when body temperature exceeds 37.8 °C) after OHCA (NCT02908308) while a second RCT will compare two different rewarming strategies (0.25 °C/h vs. 0.50 °C/h) in OHCA patients treated with TTM at 33 °C for 24 h (NCT02555254). Using large databases, reports on the specified aspects comprising the quality of the delivered therapy will help to better understand how each of the components may influence patient trajectories and how the clinical practice of TTM can be improved.

## Data Availability

Not applicable.
